# Supplementary material for: Optimal minimal residual disease threshold in pediatric acute myeloid leukemia: A retrospective cohort study based on the TARGET database
Source: PLoS Med. 2026 May 8;23(5):e1005088. doi: 10.1371/journal.pmed.1005088 (PMC13155632; doi:10.1371/journal.pmed.1005088)
Supplement: S1 Code — (ZIP) [file pmed.1005088.s002.zip › S2 code/PROJ8_16_tbl2/PROJ8_16_tbl2_seg2_2.pdf]

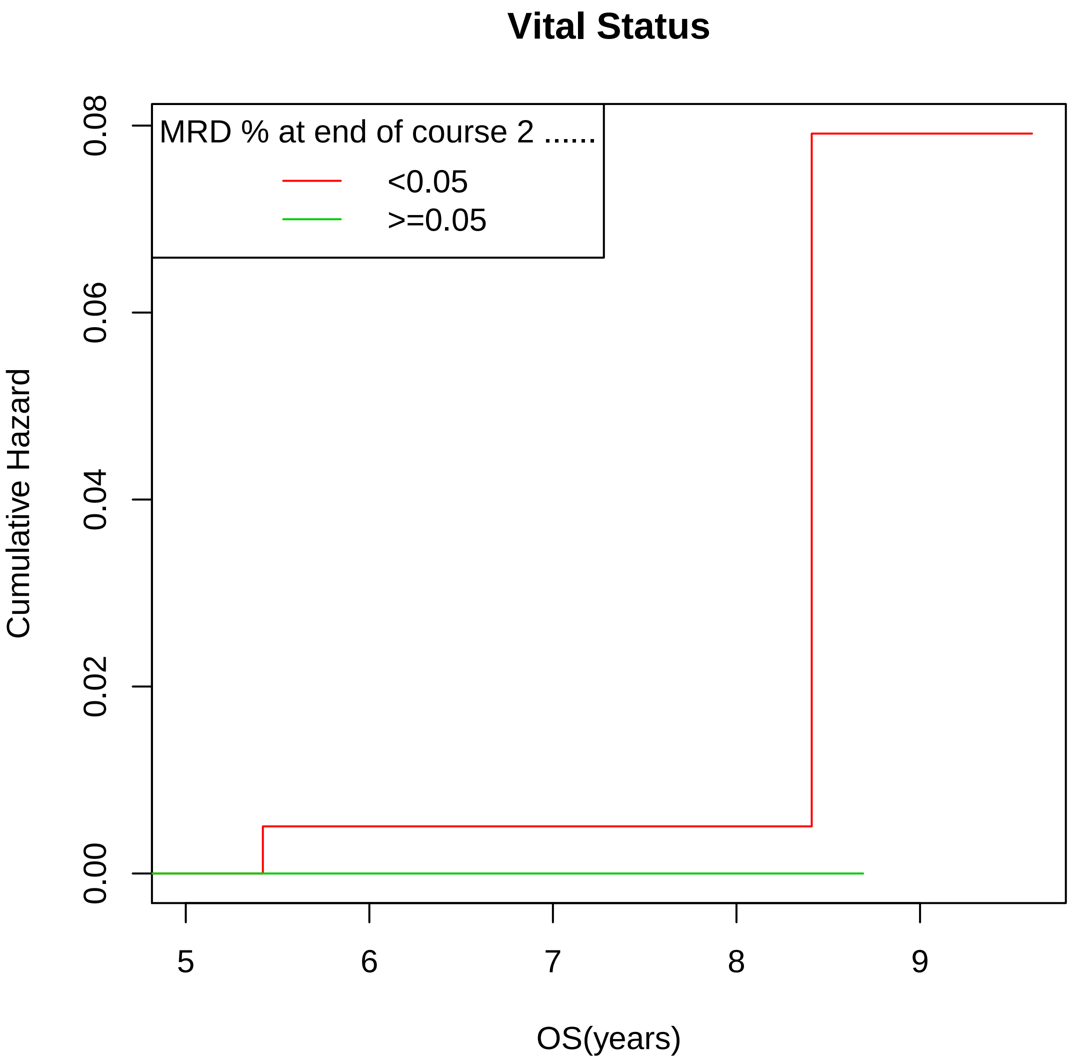

Vital Status
MRD % at end of course 2 ......
<0.05
>=0.05
Cumulative Hazard
0.00
0.02
0.04
0.06
0.08
5
6
7
8
9
OS(years)
